# Exploring Antibacterial Properties of Marine Sponge-Derived Natural Compounds: A Systematic Review

**DOI:** 10.3390/md23010043

**Published:** 2025-01-16

**Authors:** Cintia Cristina Santi Martignago, Camila de Souza Barbosa, Homero Garcia Motta, Beatriz Soares-Silva, Erica Paloma Maso Lopes Peres, Lais Caroline Souza e Silva, Mirian Bonifácio, Karolyne dos Santos Jorge Sousa, Amanda Sardeli Alqualo, Júlia Parisi, Olivier Jordan, Ana Claudia Muniz Renno, Anna Caroline Campos Aguiar, Viorica Patrulea

**Affiliations:** 1 Department of Bioscience, Federal University of São Paulo, Santos 11015-020, SP, Brazil; csantimartignago@yahoo.com.br (C.C.S.M.); homero.gmotta@outlook.com (H.G.M.); beatrizsoares.eng@gmail.com (B.S.-S.); ericapalomalopes@gmail.com (E.P.M.L.P.); lais.caroline23@unifesp.br (L.C.S.e.S.); mirian.bonifacio@unifesp.br (M.B.); karolyne.sousa@unifesp.br (K.d.S.J.S.); amanda.alqualo@gmail.com (A.S.A.); acmr_ft@yahoo.com.br (A.C.M.R.); 2 Institute of Pharmaceutical Sciences of Western Switzerland (ISPSO), University of Geneva, 1206 Geneva, Switzerland; olivier.jordan@unige.ch; 3 School of Pharmaceutical Sciences, University of Geneva, 1206 Geneva, Switzerland; 4 Department of Microbiology Immunobiology and Parasitology, Paulist School of Medicine, Federal University of São Paulo, São Paulo 04023-062, SP, Brazil; barbosa.camila12@unifesp.br (C.d.S.B.); caroline.aguiar@unifesp.br (A.C.C.A.); 5 Department of Physiotherapy, Metropolitan University of Santos (UNIMES), Santos 11045-001, SP, Brazil; juliaparisi@outlook.com

**Keywords:** antibacterial, marine sponge, multidrug-resistant, natural product, *P. aeruginosa*

## Abstract

The rise in multidrug-resistant (MDR) bacteria has prompted extensive research into antibacterial compounds, as these resistant strains compromise current treatments. This resistance leads to prolonged hospitalization, increased mortality rates, and higher healthcare costs. To address this challenge, the pharmaceutical industry is increasingly exploring natural products, particularly those of marine origin, as promising candidates for antimicrobial drugs. Marine sponges, in particular, are of interest because of their production of secondary metabolites (SM), which serve as chemical defenses against predators and pathogens. These metabolites exhibit a wide range of therapeutic properties, including antibacterial activity. This systematic review examines recent advancements in identifying new sponge-derived compounds with antimicrobial activity, specifically targeting *Pseudomonas aeruginosa*, a prevalent Gram-negative pathogen with the highest incidence rates in clinical settings. The selection criteria focused on antimicrobial compounds with reported Minimum Inhibitory Concentration (MIC) values. The identified SM include alkaloids, sesterterpenoids, nitrogenous diterpene, and bromotyrosine-derived derivatives. The structural features of the active compounds selected in this review may provide a foundational framework for developing new, highly bioactive antimicrobial agents.

## 1. Introduction

The emergence of multidrug-resistant (MDR) bacterial strains needs an extensive investigation into antibacterial compounds due to their impact on the treatment of once-effective drugs, leading to prolonged hospitalization periods, increased mortality rates, and higher healthcare costs [1]. The Infectious Disease Society of America identifies six primary MDR pathogens: *Enterococcus faecium*, *Staphylococcus aureus*, *Klebsiella pneumoniae*, *Acinetobacter baumannii*, *Enterobacter* spp., and *Pseudomonas aeruginosa*. *P. aeruginosa*, an opportunistic Gram-negative bacterium, is responsible for severe infections among hospitalized patients [2,3]. This pathogen has numerous virulence factors and adaptive mechanisms, such as biofilm formation, contributing to its resistance against multiple antibiotic classes [4]. Currently, limited pharmacological options exist for addressing *P. aeruginosa*’s resistance [5,6].

In recent decades, the pharmaceutical industry has increasingly focused on sourcing new drugs from marine origin [7]. These natural products are a valuable source of a diverse array of biologically active compounds [8,9]. Collaborative efforts between academia and industry have led to the commercialization of a few marine-derived drugs [10], including ziconotide (Prialt^®^), for severe chronic pain treatment. A recent literature review shows that the US Food and Drug Administration (FDA) has approved 15 pharmaceutical products derived from marine products for treating different conditions such as cancer, viral infections, pain, and hypertriglyceridemia. In addition, it is noteworthy that an additional 33 compounds are currently in phase I, II, or III of clinical pharmaceutical development [11].

Marine organisms with the potential to produce bioactive compounds include bacteria, fungi, microalgae, mollusks, and marine sponges (MS) [12]. Among these, MS are the most extensively studied due to their evolution of morphological and chemical defenses, primarily secondary metabolites, in response to environmental pressures like competition for resources [8,13,14]. These secondary metabolites can possess a wide range of therapeutic properties, such as anti-inflammatory, antiviral, antioxidant, anti-tumor, and antibiotic [7,11,13,15].

Given the growing need to develop new antibacterial drugs due to increased MDR rates jeopardizing the achievement of the World Health Organization (WHO)’s 2030 sustainable development goals and considering the marine environment as a potent source of new therapeutic compounds, it is essential to identify effective agents against *P. aeruginosa*, a bacterium classified as “critical” by the WHO. Therefore, the aim of this systematic literature review was to identify compounds isolated from MS that show antimicrobial activity (AA) against *P. aeruginosa.*

## 2. Results and Discussion

**Figure 1:**
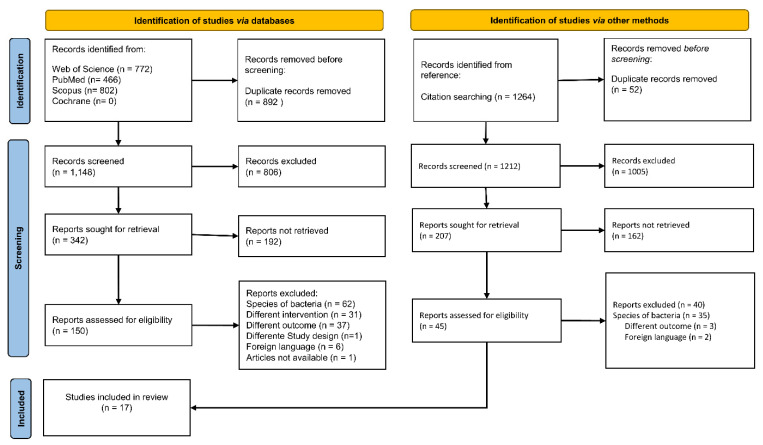
PRISMA Flow diagram of the search strategy used in the present study.

The flow diagram illustrates the search strategy used in this present study. Figure 1 shows the procedural steps of inclusion and exclusion of articles in this systematic review (SR). This process was carried out in two different phases, described in detail in the methodology section. In the first phase, a total of 2040 articles were retrieved from the following databases: Web of Science (772); Pubmed (466); and Scopus (802). Duplicates were excluded (n = 892), leaving 1148 articles for evaluation. A total of 806 articles were discarded based on the title assessment, resulting in 342 articles for further evaluation. Subsequently, 192 articles were excluded by abstract analysis, yielding 150 articles for comprehensive reading. Of these 150 papers, the following were excluded for not meeting the inclusion criteria: 62 papers focused on bacterial species other than *P. aeruginosa*; 31 for the intervention discrepancies; 37 for the outcome issues; 1 study for design inadequacy; 6 for language other than English; and 1 study unavailability. In this context, 12 articles were included at this first stage. These articles included in the first stage underwent reference evaluation for inclusion eligibility.

In the second phase, 1264 studies were evaluated, and 52 were excluded due to their duplication. The title review led to 1005 articles, leaving 207 articles for evaluation. From these, 162 studies were excluded based on abstract reading. A total of 45 articles were fully assessed, with 35 articles excluded for carrying out research with species of bacteria other than *P. aeruginosa*, 3 articles were excluded for not meeting the inclusion criteria for outcome, and 2 articles were excluded for language issues. Thus, 17 articles were included in this review.

Table 1 summarizes the data on collection locations, the specifications of the MS (genus, subclass, order, and family), and the description of all the compounds extracted and identified in the articles included in this review.

The most frequent countries for sponge collection were Brazil [16,17,18] and Mexico, [19,20,21] followed by India [22,23], Korea [24,25], China [26,27], and Australia [28,29]. Additional collection sites included the Bahamas [30], Italy [31], and the Philippines [32].

The studies included in this SR identified compounds isolated from various classes of MS, with *Heteroscleromorpha* being the most studied [16,17,18,19,20,21,24,25,26,30,32], followed by *Verongimorpha* [22,28,29], and, finally, *Keratosa* [23,27,31].

It can be seen that five of the sponges belonged to the *Agelasida* order [18,20,21,26,30], and the *Dictyoceratida* order [23,27,31] and *Verongiid* order [22,28,29] were reported in three studies each. The *Suberitida* order [24,25] appeared in two studies, whereas *Clionaida* [19], *Haplosclerida* [16], *Bubarida* [17], and *Desmacellida* [32] were reported in one study each.

Regarding the sponge genus *Spongosorites* sp. [24,25] and *Suberea ianthelliformis* [28,29] were the most frequently studied, with two articles each. In contrast, *Agelas longissima* [30], *Arenosclera brasiliensis* [16], *Psammaplysilla purpurea* [22], *Dysidea granulosa* [23], *A. mauritiana* [26], *Sphechiopongia vesparia* [19], *Dysidea avara* [31], *Petromica citrina* [17], *Agela dilatata* [20], *Desmacella* sp. [32], *A. citrina* [21], *A. dispar* [18], and the *Phyllospongia foliascens* [27] have been reported in one study each.

Table 1 shows that 98 compounds were isolated, of which 58 were tested for antibacterial activity against Gram-negative bacterium *P. aeruginosa*, as summarized in Table 2.

Supplementary Data (Table S1) provide details on the extraction and purification methods used for the compounds extracted from MS analyzed in this SR. Methanol (MeOH; CH_3_OH) was the predominant solvent used for extracting the evaluated compounds in five articles [18,22,24,25,30], while ethanol (EtOH) [17,26] and ethyl acetate (EtOAc) [23] were used in two and one study, respectively. Some researchers used solvent mixtures, with dichloromethane (DCM; CH_2_Cl_2_) and MeOH being the most common, appearing in four studies [20,21,28,29] and MeOH with EtOH or DCM carried out in single studies [16,27]. One article reported using Tris·HCl as the reagent for extraction [19] and two articles lacked specific extraction details [31,32].

**Table 1:** Description of the collection site, sponge genus, and identified compounds extracted.

Ref.	Material Collection Location	Sponge Genus Subclass/Order/Family	Compounds Extracted
[30]	San Salvador Island, Bahamas	*A. longissima* *Heteroscleromorpha*/*Agelasida*/*Argasidae*	3,7-Dimethylisoguanine and Longamide
[16]	João Fernandinho Beach, Brazil	*A. brasiliensis* *Heteroscleromorpha*/*Haplosclerida*/*Callyspongiidae*	Arenosclerins A–C and Haliclonacyclamine E
[22]	Mandapam, Tamil Nadu, India	*P. purpurea* *Verongimorpha*/*Verongiida*/*Pseudoceratinidae*	Purpurealidin A–H, Purealidin Q, 16-Debromoaplysamine-4 and Purpuramine I
[24]	Jeju Island, Korea	*Spongosorites* sp. *Heteroscleromorpha*/*Suberitida*/*Halicondrídea*	(*R*)-6″-Debromohamacanthin A; (*R*)-6′-Debromohamacanthin; (*S*)-6″-Debromohamacanthin B; *Trans*-3,4-Dihydrohamacanthin A: *Cis*-3,4-Dihydrohamacanthin B; Dibromodeoxytopsentin; Topsentin; Bromotopsentin; Eoxytopsentin; Bromodeoxytopsentin; Isobromodeoxytopsentin
[25]	Jeju Island, Korea	*Spongosorites* sp. *Heteroscleromorpha*/*Suberitida*/*Halicondrídea*	(*S*)-6′,6″-Didebromohamacanthin A; (*R*)-6′-Debromohamacanthin B; (*R*)-6′,6″-Didebromohamacanthin B; (*R*)-6″-Debromohamacanthin B (3*S*,5*R*)-6′,6″-Didebromo-3,4-dihydrohamacanthin B; Spongotine A; Spongotine B; Spongotine C; (*S*)-Hamacanthin A; (*S*)- Hamacanthin; (3*S*,5*R*)-6″-Debromo-3,4-dihydrohamacanthin B; (3*S*,6*R*)-6′-Debromo-3,4-dihydrohamacanthin A
[23]	Lakshadweep islands, India	*D. granulosa* *Keratosa*/*Dictyoceratida*/*Dysidea*	2-(2′,4′-Dibromophenyl)-4,6-dibromophenol
[26]	Paracel Islands, China	*A. mauritiana* *Heteroscleromorpha*/*Agelasida*/*Agelasidae*	(−)-Ageloxime D; (−)-8′-Oxo-agelasine D; Ageloxime B; (+)-2-Ooxo-agelasidine C 4-Bromo-*N*-(butoxymethyl)-1H-pyrrole-2-carboxamide
[28]	Manta Ray Bommie, Austrália	*S. ianthelliformis* *Verongimorpha*/*Verongiida*/*Aplysinellidae*	Ianthelliformisamine A–C; Aplysamine 1; Araplysillin I; Tokaradine C; Spermatinamine
[19]	Quintina Roo, México	*S. vesparia* *Heteroscleromorpha*/*Clionaida*/*Clionaidae*	Svl1 and Svl2
[31]	Naples, Italy	*D. avara* *Keratosa*/*Dictyoceratida*/*Dysidea*	Avarol
[17]	Xavier Island, Brazil	*P. citrina* *Heteroscleromorpha*/*Bubarida*/*Bemanthudae*	Halistanol sulfate and Halistanol sulfate C
[20]	Cozumel Island, México	*A. dilatata* *Heteroscleromorpha*/*Agelasida*/*Agelasidae*	Ageliferin; Bromoageliferin; Dibromoageliferin; Sceptrin; Nakamuric acid; 4-Bromo-1H-pyrrole-2-carboxylic acid; 4,5-Dibromopyrrole-2-carboxylic acid; 3,7-Dimethylisoguanine
[32]	Vinapor, Carmen, Philippines	*Desmacella* sp. *Heteroscleromorpha*/*Desmacellida*/*Desmacellidea*	Aaptamine, Isopentylamine, Tyramine
[21]	Cozumel Island, Mexico	*A. citrina* *Heteroscleromorpha*/*Agelasida*/*Agelasidae*	(+)-8-Epiagelasine T; (+)-10-Epiagelasine B; (+)-12-Hydroxyagelasidine C; (+)-Ent-agelasine F; (+)- Agelasine B; (+)-Agelasidine C.
[18]	Ilha do Meio, Brazil	*A. dispar* *Heteroscleromorpha*/*Agelasida*/*Agelasidae*	Disparamides A−C; Dispyrins B−F; H, H2 and H3 nagelamide; Citrinamine B; Ageliferin, Bromoageliferin, Dibromoageliferin
[27]	Woody Island, China	*P. foliascens* *Keratosa*/*Dictyoceratida*/*Thorectidae*	Phyllospongianes A−E and 12-Deacetylscalaradial
[29]	Manta Ray Bommie, Australia	*S. ianthelliformis* *Verongimorpha*/*Verongiida*/*Aplysinellidae*	Ianthelliformisamine A−C

Table 2 presents a comprehensive listing of 58 compounds isolated from MS, each of which underwent evaluation for AA as documented in the studies included in this review. Among the 17 studies incorporated in this review, 13 used a reference drug as a positive control (PC) [17,20,21,22,24,25,26,27,28,29,30,31,32], while 3 studies used more than one drug as a PC [24,29,31] and 4 studies did not provide a description regarding the use of a PC group [16,18,19,23]. Ciprofloxacin was the most frequently used drug as a PC, appearing in 5 studies, [17,26,28,29,32] followed by Meropenem and Imipenem, each used in 3 studies [20,21,24,25,29], whereas Streptomycin was used in 2 studies [22,31] and other drugs; namely Levofloxacin, Tobramycin, and Ampicillin, were used in only one study each [27,29,31]. The use of a PC in studies assessing the therapeutic efficacy of novel compounds is strongly indicated, as it facilitates the comparative analysis of outcomes with established reference drugs.

In the context of the evaluation of minimum inhibitory concentration (MIC), a total of nine publications failed to describe the percentage of inhibition evaluated [16,17,22,24,25,27,29,30,31]; five studies reported complete growth inhibition [20,21,27,29,32]; four studies reported 50% growth inhibition [19,23,26,28], and one study reported an inhibition concentration of 90% [23]. It is important to mention that both Xu et al. 2012 and Shrindhar et al. 2009 carried out their evaluations at two different inhibition percentages [23,28].

Concerning bacterial strains of *P. aeruginosa* used in the studies included in this review, it is important to highlight that six studies did not provide specifications regarding the strains used [16,22,23,27,28,30], whereas seven studies used *P. aeruginosa* ATCC 27853 strains [17,18,19,20,21,26,31]. The strains PAO1 and 1771M were each used in two studies [20,24,25,29]; the strains 1592E and 1771 were used in the study conducted by Bao et al. 2007 [25], while the strain BIOTECH 1335 was used in the study of Mazo et al. 2022 [32].

Regarding the compounds isolated from MS that were analyzed in this study, it is noteworthy to highlight that 36 demonstrated inhibitory activity against *P. aeruginosa*, whereas 21 did not show such inhibition. It is particularly interesting that compounds **27** and **28** presented different results in the studies run by Xu et al. 2012 and Tran et al. 2023, with this divergence in results attributable to the difference in percentages of inhibition that were evaluated. Compounds **42**–**44** showed different results when evaluated against other bacterial strains. Although these three compounds showed inhibitory activity against *P. aeruginosa* ATCC 27853, this activity was not maintained when evaluated against *P. aeruginosa* PAO1, demonstrating a degree of selectivity in their AA.

Compounds **1**–**3**, **29**, **37**, **42**–**44**, and **56** showed inhibition activity at a concentration above 51 μg/mL, while compounds **5**, **10**–**21**, **38**–**39**, **52**, and **58** demonstrated inhibition at lower concentrations between 10 and 50.9 μg/mL. Conversely, compounds **4**, **32**–**34**, **46**–**47**, **54**–**55**, and **57**, displayed bacteria inhibition at concentrations below 9.9 μg/mL. It is important to note that compounds **37**–**39**, **52**, and **29** showed complete inhibition of bacterial growth at concentrations between 10 and 70 μg/mL, while compounds **46**–**47** achieved similar results at concentrations below 9 μg/mL. Furthermore, it is important to note that this activity was evaluated at different percentages of inhibition, which are described in detail in Table 2.

In contrast, compounds **22**–**26** did not exhibit any inhibitory effects on the bacteria when assessed at the threshold of fifty percent inhibition. The compounds **40**–**42**, **45**, **48**–**51**, and **53** failed to obtain complete growth inhibition. Additionally, the compounds that did not show activity against *P. aeruginosa*, for which the authors did not describe the evaluated percentage of inhibition, included compounds **6**–**9**, **30**, **35**, and **36**.

**Table 2:** Description of the percentage of inhibition concentrations of the evaluated isolated compounds.

Ref.	Compounds (Compounds Number)	MIC (%)	Bacteria Strains	Results
[30]	Longamide (**1**)	ND	ND	>60 μg/mL
[16]	Haliclonacyclamine E (**2**) Arenosclerins A (**3**) Arenosclerins B (**4**) Arenosclerins C (**5**)	ND	ND	200 μg/mL 400 μg/mL 5 μg/mL 50 μg/mL
[22]	Purealidin Q (**6**) Purpurealidin B (**7**) 16-Debromoaplysamine-4 (**8**) Purpuramine I (**9**) * Steptomycin	ND	ND	NAA NAA NAA NAA 10 μg/mL
[24]	(*R*)-6″-Debromohamacanthin A (**10**) (*R*)-6′-Debromohamacanthin A (**11**) (*S*)-6″-Debromohamacanthin B (**12**) *Trans*-3,4-Dihydrohamacanthin A (**13**) *Cis*-3,4-Dihydrohamacanthin B (**14**) * Imipenem * Meropenem	ND	1771 M	25 μg/mL 25 μg/mL 25 μg/mL >25 μg/mL 25 μg/mL 0.098 μg/mL 0.098 μg/mL
[25]	(*S*)-6′,6″-Didebromohamacanthin A (**15**) (*R*)-6′-Debromohamacanthin B (**16**) (*R*)-6′,6″-Didebromohamacanthin B (**17**) (3*S*,5*R*)-6″-Debromo-3,4-dihydrohamacanthin B (**18**) (3*S*,6*R*)-6′-Debromo-3,4-dihydrohamacanthin A (**19**) Spongotine B (**20**) (3*S*,5*R*)-6′-Debromo-3,4-dihydrohamacanthin B (**21**) * Meropenem	ND	1592 E; 1771; 1771 M;	25/25/>25 μg/mL >25/25/25 μg/mL 25/25/25 μg/mL 25/25/25 μg/mL 25/25/25 μg/mL >25/>25/25 μg/mL >25/>25/25 μg/mL 0.098/0.391/0.049
[23]	2-(2′,4′-Dibromophenoxy)-4,6-dibromophenol (**22**)	50	ND	NAA
		90		NAA
[26]	(−)-Ageloxime D (**23**) (−)-8′-Oxo-agelasine D (**24**) (−)- Ageloxime B (**25**) (+)-2-Oxo-agelasidine C (**26**) * Ciprofloxacin	50	ATCC 27853	NAA NAA NAA NAA --
[28]	Ianthelliformisamine A (**27**) Ianthelliformisamine B (**28**) Ianthelliformisamine C (**29**) Aplysamine 1 (**30**) Araplysillin I (**31**) * Ciproflaxacin	50	PAO1	6.8 μM NAA 8.9 μM NAA NAA 0.038 μM
	Ianthelliformisamine A (**27**) Ianthelliformisamine B (**28**) Ianthelliformisamine C (**29**) Aplysamine 1 (**30**) Araplysillin I (**31**)	ND	PAO1	35 μM 87.5 μM 17.5 μM NAA 175 μM
[19]	Svl 1 (**32**) Svl 2 (**33**)	50	ATCC 27853	3.90 μg/mL 3.90 μg/mL
[31]	Avarol (**34**) * Streptomycin * Ampicillin	ND	ATCC 27853	3 μg/mL 170 μg/mL 740 μg/mL
[17]	Halistanol sulfate (**35**) Halistanol sulfate C (**36**) * Ceftazidime	ND	ATCC 27853	NAA NAA 30 μg
[20]	Ageliferin (**37**) Bromoageliferin (**38**) Dibromoageliferin (**39**) Sceptrin (**40**) Nakamuric acid (**41**) 4-Bromo-1H-pyrrole-2-carboxylic acid (**42**) 4,5-Dibromopyrrole-2-carboxylic acid (**43**) 3,7-Dimethylisoguanine (**44**) * Imipenem	Complete growth inhibition	ATCC 27853/PAO1	64/64 μg/mL 8/32 μg/mL 32/32 μg/mL NAA/NAA μg/mL NAA/NAA μg/mL 64/NAA μg/mL 64/NAA μg/mL 64/NAA μg/mL 2/2 μg/mL
[32]	Aaptamine (**45**) Isopentylamine (**46**) Tyramine (**47**) * Ciprofloxacin	Complete growth inhibition	BIOTECH 1335	NAA 1.56 μg/mL 8.85 μg/mL 6.77 μg/mL
[21]	(+)-8-Epiagelasine T (**48**) (+)-10-Epiagelasine B (**49**) (+)-12-Hhydroxyagelasidine C (**50**) (+)-Ent-agelasine F (**51**) (+)-Agelasine B (**52**) (+)-Agelasidine C (**53**) * Imipemen	Complete growth inhibition	ATCC 27823	NAA NAA NAA NAA 16 μg/mL NAA ND
[18]	Ageliferin A (**37**) Ageliferin B (**38**) Dibromoageliferin (**39**)	Complete growth inhibition	ATCC 27853	50 μM 12.5 μM 25 μM
[27]	Phyllospongiane A (**54**) Phyllospongiane B (**55**) Phyllospongiane C (**56**) Phyllospongiane D (**57**) Phyllospongiane E (**58**) * Levofloxacin	ND	ND	4 μg/mL 4 μg/mL >64 μg/mL 4 μg/mL 32 μg/mL 0.25 μg/mL
[29]	Ianthelliformisamine A (**27**) Iantheliformisamine B (**28**) Ianthelliformisamine C (**29**) * Ciprofloxacin * Tobramycin * Meropenem	Complete growth inhibition	PAO1	NAA NAA 53.1 μg/mL 0.25 μg/mL 2 μg/mL 0.375 μg/mL

NAA—No Antimicrobial Activity; ND: Not Described; *: Positive Control; ATCC: American Type Culture Collection.

**Figure 2:**
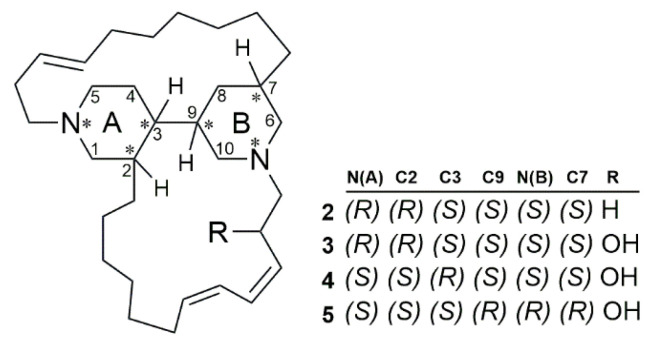
General structure of tetracyclic alkylpiperidine alkaloids. The * indicates the chiral center in the molecule.

All tetracyclic alkylpiperidine alkaloids **2**–**5** reported by Torres et al. (2002) displayed antibacterial activity against *P. aeruginosa*. Compounds **4** and **5** exhibited higher antibacterial activity compared to compounds **2** and **3.** Compounds **2** and **3** showed similar MICs (200 and 400 µg/mL, respectively), suggesting that the hydroxy group present in compound **3** does not significantly contribute to antibacterial activity. Additionally, the data reported indicate that the stereochemical configuration of the bispiperidine ring system is important for the inhibitory activity within this series. Compounds **3**, **4**, and **5** exclusively differ in the configuration of the nitrogen and carbon atoms at positions 2, 3, 7, and 9 (Figure 2). This difference results in an 80-fold increase in antibacterial activity for compound **4** and an 8-fold increase for compound **5**, compared to compound **3**.

**Figure 3:**
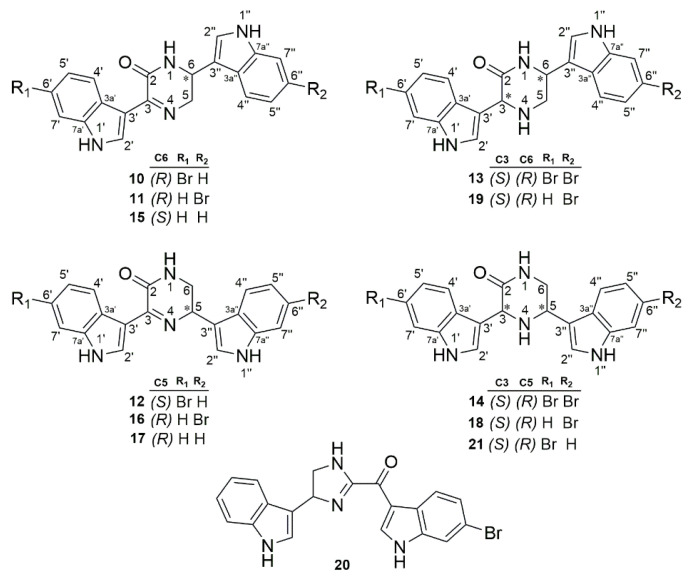
General structures of bisindole alkaloids. The * indicates the chiral center in the molecules.

In 2005 and 2007, Bao et al. reported the identification and antibacterial activity of bisindole alkaloids (Figure 3) isolated from MS [24,25]. The biological evaluation of compounds **10**, **11**, and **15**, which belong to the hamacanthin A class, suggests that bromine at positions R1 or R2 improves antibacterial activity against *P. aeruginosa* 1771M (Table 2). Moreover, the presence of an additional chiral center in carbon 3 appears to be irrelevant as it decreased the inhibitory effect of compound **19** in comparison to compound **11**. Similar effects are identified in compounds belonging to the hamacanthin B class, where the presence of bromine in compounds **12** and **16** improved activity, while the addition of another chiral center reduces it (when comparing compounds **16** and **18**). Interestingly, the presence of two bromine atoms in compound **14** significantly improved its inhibitory effect, a result not replicated in compound **13**. Finally, substituting dihydropiperazinone or piperazinone with dihydroimidazole was not successful. Furthermore, compounds **15**–**21** were evaluated against three different *P. aeruginosa* strains, showing different effects for each strain.

**Figure 4:**
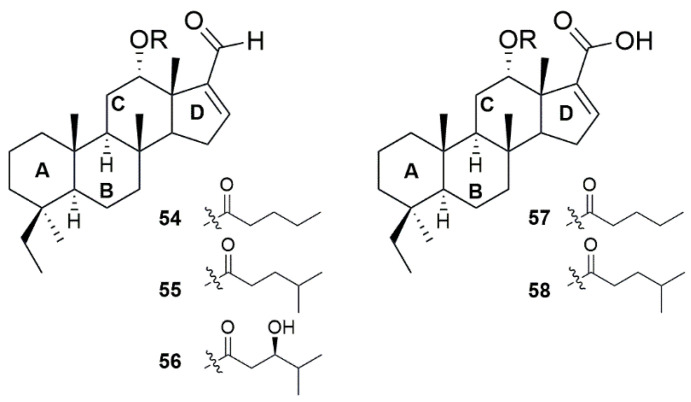
The general structure of scalarane alkaloids.

In 2023, Yu and colleagues reported the isolation of five new scalaranes (Figure 4) that displayed spectral profiles different from the known scalarane sesterterpenoids [27]. Interestingly, instead of a 6/6/6/6-tetracyclic fused ring system, these compounds possess a 6/6/6/5-tetracyclic dinorscalarane scaffold. These new chemical scaffolds showed promising antibacterial activity, with compounds **54**, **55**, and **57** exhibiting MIC values of 4 µg/mL each. For compounds containing the valerate group (**54** and **57**), the presence of a carboxylic group at *D*-ring significantly improved antibacterial activity by more than 16-fold. The substitution of the valerate group with a 4-methylpentanoate group (compounds **57** and **58**) resulted in lower inhibitory effect against *P. aeruginosa* by 13-fold. On the other hand, this same substitution in compounds containing a ketone in the *D*-ring (compounds **54** and **55**) resulted in an improved potency exceeding 16-fold. Interestingly, the addition of a hydroxy group in the side chain (**56**) had a poor effect on antibacterial potency.

**Figure 5:**
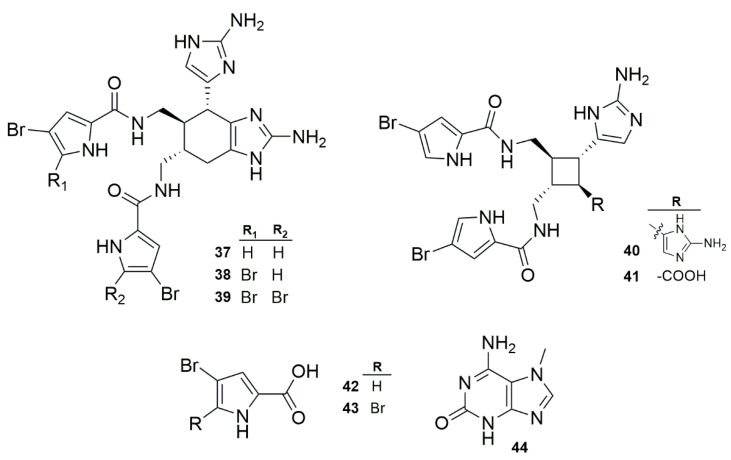
The general structure of bromopyrrole and imidazole-containing alkaloids.

Freire et al. 2022 [18] and Pech-Puch et al. 2020 [20] reported the antibacterial effect of bromopyrrole alkaloids derived from the oroidin family, which were isolated from sponges belonging to the genus *Agelas* (Figure 5). Both studies showed three ageliferins with promising antibacterial effects against *P. aeruginosa* (ATCC 27853), with compounds **38**, **39**, and **37** displaying high inhibitory effects (8, 32 and 64 mg/mL, respectively). These data suggest that the presence of two bromines in at least one pyrrole ring may enhance antibacterial activity. Interestingly, compounds **40** and **41**, which both contained cyclobutane, due to a different cyclization process, led to compounds with no antibacterial effect (MIC ≥ 128 mg/mL) against *P. aeruginosa*. Moreover, compounds **42**, **43**, and **44** showed low inhibitory effects; because of their low molecular weight, they could represent interesting backbones for further exploration in antibacterial research.

**Figure 6:**
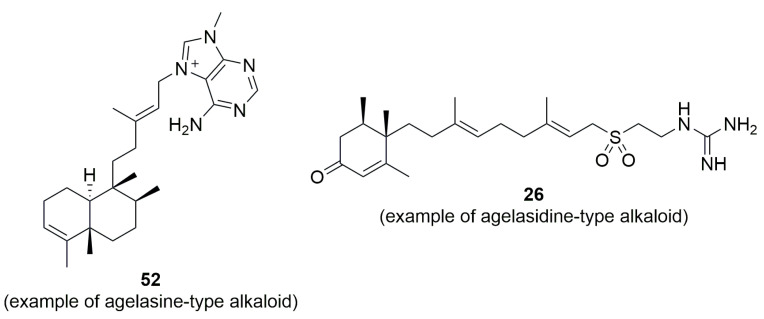
Example of agelasine- and agelasidine-type alkaloids isolated from *Agelas* sponges.

Yang et al. and Pech-Puch et al. reported the isolation of nitrogenated diterpene agelasine- and agelasidine-based alkaloids (Figure 6), which are derived from MS of the *Agelas* genus [21,26]. In general, the derivatives of agelasine and agelasidine showed a lack of AA against *P. aeruginosa*, with the exception of compound **52**, which exhibited a MIC value of 16 µg/mL. Conversely, these compounds seemed to be effective against Gram-positive bacteria.

**Figure 7:**
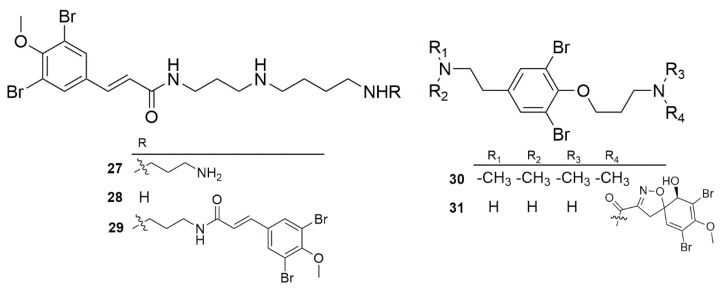
The general structure of ianthelliformisamine compounds.

Bromotyrosine-derived metabolites (Figure 7) isolated from *Suberea* sponges were active against *P. aeruginosa*. Xu et al. reported that compounds **27** and **29** were able to inhibit bacterial growth at 6.8 µM (3.5 µg/mL) and 8.9 µM (7.5 µg/mL) against the *P. aeruginosa* PAO1 strain, respectively [28]. Interestingly, Tran and colleagues (2023) showed that compound **29** inhibited the growth of free-living PAO1 bacteria at 53.1 µg/mL, which could be a consequence of different experimental conditions. Additionally, the latter study revealed that this compound also inhibited biofilm formation, while compounds **27** and **28** did not show any inhibitory effect against free-living bacteria and failed to prevent biofilm formation. Surprisingly, compounds **27** and **28** exhibited synergistic interactions with ciprofloxacin, a known antibiotic, while such an interaction was not seen for compound **29**. This synergistic effect aligns with previous reports suggesting that ianthelliformisamines serve as antibiotic enhancers [33].

Although the goal of this SR was to focus on *P. aeruginosa*, compounds extracted from MS have been widely used on more than one bacteria strain, and these results are detailed in the Supplementary Data (Figure S1).

Ten articles included in this review also analyzed other therapeutic properties of the compounds listed in the table below (Table 3). Among the analyses carried out, the most frequent was cytotoxicity, which was evaluated in 6 articles [16,18,24,25,27,29], followed by antifungal properties, evaluated in 2 articles [22,31]. Additionally, antileishmanial, anti-inflammatory, antidiabetic, and hemolytic activities were each evaluated in one study [18,26,32].

The compounds **2**–**5**, **10**–**21**, **27**–**29**, and **37**–**38** had their cytotoxic activity evaluated against different cell lines (Table 3); specifically compounds **27**–**28** showed low cytotoxicity in human embryonic kidney cells, while compounds **37**–**39** showed no cytotoxicity in OVCAR3 cells. All remaining compounds evaluated showed IC50 at the different concentrations tested.

Regarding the results of the antifungal analyses, we can highlight that although the compounds **6**–**9** have not shown an antifungal effect against *Aspergillus fumigatus*, *Fusarium* spp., *Cryptococcus neofromans*, *A. niger*, *Rhodotorula* sp., *Norcardia* sp., *Candida albicans*, compound **34** showed significant activity against *A. fumigatus*, *A. versicolor*; *A. ochraceus*, *A. niger*, *Trichoderma viride*, *Penicillium funiculosum*, *P. ochrochloron*, and *P. verrucosum* var. *cyclopium*.

The compounds **37**–**39** showed no hemolytic activity, while compounds **45**–**47** showed not only anti-diabetic activity, but also anti-inflammatory activity. Additionally, antileishmanial activity was demonstrated by compounds **23** and **25**.

It is worth noting that most of the compounds subjected to evaluation of their antibacterial activity were concurrently assessed for their toxicity profiles. Compounds **2**–**5**, **10**–**21**, and **54**–**59** showed both antimicrobial and toxicity activity across different cancer cell lines, demonstrating a dual potential for antibacterial and anticancer therapeutic activities. Although compounds **37**–**39** showed AA, they did not show toxicity in the cancer cell lines evaluated. Compounds **27**–**29** displayed low toxicity in normal cell lines; however, only compound **28** had antibacterial activity.

**Figure 8:**
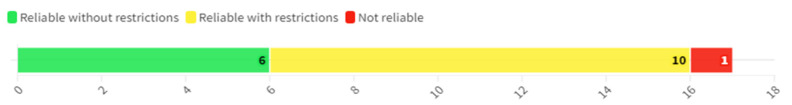
Results of the risk of bias analysis of the studies included in this review.

All articles included in the review were assessed for their risk of bias according to the categorization criteria of the ToxRTool^®^, a Microsoft^®^ Office Excel spreadsheet-based tool publicly available. The results are summarized in Figure 8 and described in detail in the Supplementary Data (Table S2).

The assessment of bias risk revealed that out of 17 articles included in this review, 6 were categorized as “reliable without restrictions” as they provided sufficient data to demonstrate adherence to the established criteria.

A total of 10 out of the 17 articles [16,18,22,23,24,25,26,27,31,32] were classified as “reliable with restrictions”. In all of these articles, except for Pejin et al. (2014), the statistical analysis methodologies used for analyzing the acquired data were not mentioned [31]. Additionally, Torres et al. (2002), Tilvi et al. (2004), Bao et al. (2005), Bao et al. (2007), and Pejin et al. (2014) failed to reference the characterization tests that were used [16,24,25,31]. Tilvi et al. (2004), Bao et al. (2005), Bao et al. (2007), Shridhar et al. (2009), Yang et al. (2012), Mazo et al. (2022), Freire et al. (2022), and Yu et al. (2023) did not mention the use of negative controls in the conducted tests [18,22,24,25,26,27,31,32].

Freire et al. (2022) similarly did not provide information regarding the use of PC, and Yu et al. (2023) did not inform the number of replicates used in the tests [18,27]. Yang et al. (2012) neglected to report the doses and concentrations used, as well as the frequency and duration of exposure to the test substance in group IV of key questions [26].

One of the articles was classified as not reliable due to the absence of detailed descriptions regarding the conditions of cell culture in group I, while in group III, the responses were found to be non-compliant, as significant information was entirely lacking from the article, such as a comprehensive account of the method of administration in the culture medium, the frequency and duration of exposure, the presence of both negative and PC, as well as the number of replicates used [30].

The synthesis of scientific evidence was conducted using the GRADE approach. The summary of the evidence presented in Table S3 showed a moderate quality for studies using in vitro analysis of AA quantified by MIC values. However, certain studies failed to fully address all review questions, particularly lacking detailed statistical descriptions, methodological information, and the inclusion of negative controls in experimental procedures. A detailed analysis and justification for the downgrading of evidence quality is presented in Supplementary Materials (Table S3).

## 3. Materials and Methods

This SR was conducted following PRISMA (The Preferred Reporting Items for Systematic Reviews and Meta-Analysis) guidelines [34]. The search for relevant articles was carried out from 8 to 13 November 2023, across the following databases: Pubmed, Scopus, and Web of Science, employing the subsequent descriptors from the Medical Subject Headings (MeSH): (“Porífer*” OR “Marine Sponge” OR “Demospongia*”) AND (“Antibiotic*” OR “Antibacterial*” OR “Antimicrobial” OR “Anti-Infective Agents”), which were modified according to specific characteristics of each database. The search parameters were filtered by title and abstract. Between 4 and 11 October 2024, the dataset was updated within the same databases using the same descriptors.

The study selection was carried out individually by two reviewers, BSS and HM, with the assistance of the Rayyan software (https://www.rayyan.ai/), and consisted of two different phases, each with three stages. In the first phase, articles retrieved from databases mentioned earlier were selected for revision. To ensure comprehensive coverage of relevant studies, a second inclusion phase was carried out, in which the bibliographic references of the articles included in the first phase were also analyzed. Duplicate articles from both the first and second inclusion phases were removed before proceeding with the selection process.

The selection was performed in three stages: first, articles were screened based on their titles; second, based on their abstracts; and finally, through full-text review. This selection adhered to the inclusion and exclusion criteria outlined in Table 4. In cases where the reviewers disagreed on whether an article should be included, a third reviewer, CCSM, with extensive experience, was consulted to mediate and resolve the disagreements.

All the articles included in this study underwent a meticulous extraction and summarization of their fundamental information, which was subsequently organized into tables or visual representations. The primary data collected pertain to the sponges under investigation, with all extracted compounds systematically described in Table 1. The data relating to the antimicrobial MIC values can be found in Table 2, alongside other in vitro tests carried out within the scope of these, also presented in Table 3. Furthermore, the Supplementary Material provides detailed information regarding the assessment of the risk of bias, the methodology used for compound extraction, the outcomes of the compounds assessed against various bacterial strains, and the assessment of the quality of evidence.

Assessment of the risk of bias was carried out using the ToxRTool^®^ (Toxicological Data Reliability Assessment Tool, created by EURL-ECVAM) [35]. The reliability of data was thoroughly evaluated in the context of this investigation. Through the application of this tool, which facilitates a comprehensive assessment of the methodological quality of the included studies, a precise and reliable analysis of the result is rendered feasible. This technique also guarantees impartial and consistent analysis.

Based on answers to particular methodological questions, the ToxRTool^®^ allows the assignment of a score from 0 to 18 for each research project. The areas covered by these questions include: identification of the test substance, characterization of test system, description of study design, documentation of the study results, and evaluation of plausibility of study design and data. Every inquiry is assigned a score of either 0 or 1, indicating non-adherence to established standards of quality or compliance.

Studies scoring between 15 and 18 points, while adequately addressing all essential questions, were classified as “reliable without restrictions.” Those scoring between 11 and 14 were classified as “reliable with restrictions”, while those scoring between 0 and 10 were classified as “not reliable”.

The GRADE tool (Grading of Recommendations, Assessment, Development, and Evaluations, accessed at https://gdt.gradepro.org/app/) was used to assess the quality of the evidence. This tool evaluates all the included studies combined and classifies the quality of the evidence into four distinct categories: high (indicating sufficient evidence to estimate the effect), moderate (suggesting the true effect is likely to be close to the estimated effect), low (denoting limited confidence in the effect), and very low (reflecting minimum confidence in the estimated effect) [36].

## 4. Conclusions

This SR compiled an extensive dataset, detailing sponge-derived compounds and their antimicrobial efficacy, including minimum inhibitory concentration values, with a particular focus on the Gram-negative bacterium *P. aeruginosa* and related in vitro evaluations. Among the 98 compounds isolated from MS, only 58 were tested against *P. aeruginosa*, with 34 demonstrating bioactivity against this pathogen.

This SR accentuates the significance of meticulous data reporting of superior quality, which encompasses essential components such as methodological rigor, inhibition metrics, comprehensive statistical analyses, and the incorporation of both positive and negative control groups. These elements are crucial for discerning the most promising candidates to address antimicrobial resistance and facilitate substantive progress within the discipline. This is especially pertinent in a clinical setting, as we face the pressing imperative of fighting highly dangerous multidrug-resistant pathogens.

However, to draw definitive conclusions about these compounds, more detailed studies are needed, preferably including in vivo experiments. It is also crucial to provide clear and precise information about the experimental conditions used in each study, as most studies lack such details.

## Figures and Tables

**Table 3:** Overview of additional in vitro analyses carried out in the studies.

Ref.	Additional Assay (Compounds Tested)	Methodology	Results
[16]	Cytotoxicity (**2**–**5**)	HL-60, L929, B16 and U138 cells were evaluated for viability using the MTT.	All the compounds evaluated presented IC50 for the cells tested between the concentrations of 4.31–1.71 µg/mL.
[22]	Antifungal (**6**–**9**)	Were used strains of *A. fumigatus*, *Fusarium* spp., *C. neofromans*, *A. niger*, *Rhodotorula* sp., *Norcardia* sp., *C. albicans* for antifungal activity and Nystatin was used as a PC.	All of the compounds show absence of activity against all fungal strains.
[24]	Cytotoxicity (**10**–**14**)	A549; SK-OV-3; SK-MEL-2; XF498 and HCT15 cells were assessed. The cell culture was fixed with cold TCA and stained by sulfo-rhodamine B.	All the compounds evaluated presented IC50 for the cells tested between the concentrations of 30–2.83 µg/mL.
[25]	Cytotoxicity (**15**–**21**)	A549; SK-OV-3; SK-MEL-2: XF498 and HCT15 cells were harvested, counted, and inoculated. The culture was fixed with cold TCA and stained by sulfo-rhodamine B.	All the compounds evaluated presented IC50 for the cells tested between the concentrations of 30–3.71 µg/mL.
[26]	Antileishmanial (**23**–**26**)	Antileishmanial activity of the compounds was assessed in the culture of *Leishmania donovani* promastigotes and Pentamidine and amphotericin B were used as the PC.	Compounds **23** and **25** exhibited antileishmanial activity against *L. donovani* with IC50/IC90 values of 29.28/33.96 and 28.55/33.19 μg/mL, respectively.
[31]	Antifungal (**34**)	Strains of *A. fumigatus*, *A. versicolor; A. ochraceus*, *A. niger*, *T. viride*, *P. funiculosum*, *P. ochrochloron*, and *P. verrucosum* var. *cyclopium* were assayed for antifungal activity and ketoconazole was used as a PC.	The compound **34** exhibited antifungal activity against all fungi evaluated, with concentrations between 4 and 15 µg/mL.
[32]	Anti-inflammatory; Antidiabetic (**45**–**47**)	Anti-inflammatory activity was assessed using membrane stabilization and albumin denaturation inhibition tests. An antidiabetic assay was performed based on the starch-iodine test.	All the compounds inhibited the hemolysis of red blood cells and protein denaturation. Maximum inhibition was observed at 100 μg/mL. All compounds exhibited a meaningful α-amylase inhibitory effect.
[18]	Cytotoxicity Hemolytic Activity (**37**–**39**)	OVCAR3 cells were used for cytotoxicity assay. Erythrocytes were collected from BALB/c mice, and seeded at a 3% suspension for 2 h at 24 °C. The untreated erythrocytes were used as a negative control.	No compound showed cytotoxicity for the cell lines tested. No significant damage to the red blood cells could be observed after incubation at 100 μM.
[27]	Cytotoxicity (**54**–**58**)	MDA-MB-231, HepG2, C4−2-ENZ, MCF-7, H460, and HT-29 cells were evaluated for viability using the CCK-8.	All the evaluated compounds exhibited IC50 for the tested cells within the concentration range of 50–0.7 µM.
[29]	Cytotoxicity (**27**–**29**)	HEK293 cells were evaluated for viability using resazurin.	All the compounds induced low toxicity up to the highest testing concentrations

HL-60: leukemia cells; L929: fibrosarcoma cells; B16: melanoma cells; U138 colon cells; OVCAR3: human ovarian cancer cells; MDA-MB-231: breast cells; HepG2: liver cells; C4−2-ENZ: prostate cells; MCF-7: breast cell; H460: lung cells; HT-29: colon cell; HEK293: human embryonic kidney cells; A549: lung cell; SK-OV-3: human ovarian cancer; SK-MEL-2: melanoma cell and HCT15: colon cell; XF498: glioblastoma cell.

**Table 4:** Inclusion and Exclusion criteria.

Inclusion	Exclusion
Tested on the bacteria *P. aeruginosa*	Compounds tested on bacteria other than *P. aeruginosa*
Compounds extracted from MS without any other associated microorganisms	Compounds extracted from microorganisms associated with MS
Analysis of AA with MIC	Not evaluating AA by MIC
In vitro studies and original	Review or in vivo articles
Published in English	Published in a language other than English
Articles published between 1993–2024	Articles published before 1993
Having a control group with or without treatment	No control groups (positive or negative)

MS: marine sponge; AA: antibacterial activity; MIC: minimal inhibitory concentration.

## Data Availability

The raw data supporting the conclusions of this article will be made available by the authors on request.

## Supplementary Materials

The following supporting information can be downloaded at: https://www.mdpi.com/article/10.3390/md23010043/s1, Figure S1: Graphical representation of the compounds that were evaluated for their activity on other bacteria; Table S1: Description of the extraction and isolation protocol of the antimicrobial compounds; Table S2: Risk of bias assessment results based on ToxRTool Assessment Criteria; Table S3: Synthesis of scientific evidence: GRADE.
