# Supplementary material for: Exploring Antibacterial Properties of Marine Sponge-Derived Natural Compounds: A Systematic Review
Source: Mar Drugs. 2025 Jan 16;23(1):43. doi: 10.3390/md23010043 (PMC11766522; doi:10.3390/md23010043)
Supplement: Supplementary file 1 [file marinedrugs-23-00043-s001.zip › marinedrugs-3384065-supplementary.pdf]

Table S1 Description of the extraction and isolation protocol of the antimicrobial compounds.

Author	The method applied for extraction of compounds	The method applied for isolation compounds
[30]	The compounds were extracted with MeOH of the sample frozen.	The crude extract was partitioned between *n*-BuOH and water. The *n*-BuOH-soluble material was chromatographed by MPLC on silica gel with a solvent gradient system from EtOAc to MeOH. The fraction eluted with EtOAc/MeOH was further purified by HPLC.
[16]	The compounds were extracted in a mixture of MeOH: EtOH overnight.	The crude extract was subjected to Si gel flash chromatography. The second and fifth fractions were subjected to chromatography on a Si gel cyclopropyl-bonded LOBAR column with a gradient of MeOH in CH_2Cl_2. The whole sample was purified by chromatography on a reversed-phase C18 Sep-Pak cartridge.
[22]	The compounds were extracted with MeOH of the sample frozen.	The extract was chromatographed on a silica gel column using dichloromethane and methanol as eluent. The fraction eluted with 8% MeOH was further purified by repeated gel chromatography, Sephadex LH20, to get compound **6-7.** The fractions eluted with 10% MeOH were purified on reverse phase HPLC, which afforded compounds **8-9.**
[24]	The compounds were extracted with MeOH of the sample frozen.	The crude extract was partitioned between CH_2Cl_2 and water. The CH_2Cl_2 layer was further partitioned between aqueous MeOH and *n*-hexane. The aqueous MeOH fraction was subjected to gradient reversed-phase flash column chromatography and **16** fractions were obtained. These fractions were submitted to reversed-phase HPLC to obtain the isolated compounds.
[25]	The compounds were extracted with MeOH of the sample frozen.	The crude extract was partitioned between CH_2Cl_2 and H_2O. The CH_2Cl_2 layer was further partitioned between aqueous MeOH and *n*-hexane. The purification process was carried out on a reversed-phase HPLC, thirteen sub-fractions were obtained.
[23]	The compounds were extracted after the freeze-drying of the sample with successive baths of EtOAc and MeOH.	The EtOAc extract was chromatographed on Sephadex LH20. Fractions of similar composition were combined and purified on silica gel to obtain the single main polybrominated compound **22.**
[26]	The compounds were extracted with 95% EtOH at room temperature.	The EtOH extract was suspended in H_2O and extracted with EtOAc. The EtOAc-soluble extract was partitioned between MeOH/H_2O and petroleum ether. The MeOH/H_2O phase was diluted with additional H_2O and extracted with CH_2Cl_2 to afford the CH_2Cl_2-soluble extract, which was subjected to VLC on silica gel to give four fractions. Fraction A was separated on a Sephadex LH-20 column, resulting in five fractions (A1–A5). Sub-fraction A4 was further subjected to column chromatography using MeOH/H_2O, yielding 13 sub-fractions (A401–A413). Fraction A411 was separated by repeated CC on silica gel and followed by HPLC to yield compound **23.** Similarly, fraction A410 was repeatedly subjected to chromatography on silica gel and purified by HPLC, to afford compounds **23** and **25**. The fraction B was repeatedly subjected to CC on silica gel and further purified by HPLC to yield compound **26**.

[28]	The samples and *n*-hexane were stirred for 2 hours and then the samples were filtered, and the sponges passed on to the next baths in a CH_2Cl_2/CH_3OH for 2 hours and the resulting extract was filtered. Finally, two CH_3OH baths were carried out. The extracts were combined and dried under pressure.	The crude extract was pre-adsorbed onto C18-bonded silica and then packed into a protective stainless steel HPLC cartridge. Sixty fractions were collected, the fractions 34 and 45 contained the ions of interest and, after lyophilization, yielded compounds **27 - 29**. Fractions 23-25 were combined and purified on an HPLC column. Sixty fractions were collected, and fraction 16 yielded pure compound **30**. Fraction 39 was also further purified using HPLC, sixty fractions were collected, and fraction 26 yielded compound **31**.
[19]	The compounds were extracted from the marine sponge homogenized with 0.05 M Tris-HCl, pH 7.5, and centrifuged at 6,000 rpm for 20 min. The supernatant was dialyzed against distilled water.	The crude extract was fractionated by ammonium sulfate precipitation. The fraction with the highest hemagglutination activity was loaded to an *Octyl*-Sepharose-(NH_4) SO_4 column and eluted with a linear gradient of 0.05 M Tris-HCl-0.3 M $(NH_4)SO_4{\cdot}H_2O$. This fraction was then applied to a DEAE-Toyopearl anion-exchange column using HPLC.
[31]	*ND*	*ND*
[17]	The compounds were extracted by maceration with EtOH 92% at room temperature for three days.	The EtOH extract was redissolved in H_2O, and subsequently extracted with EtOAc and *n*-BuOH. An aliquot of the *n*-BuOH fraction was then submitted to the Sephadex LH-20 chromatography column, resulting in five sub-fractions. After further column chromatography fractionation, compounds **35** and **36** were yielded.
[20]	The compounds were extracted with exhaustive baths of CH_3OH/CH_2Cl_2	The extract was then partitioned into hexane, *n*-BuOH, dichloromethane, and a final aqueous methanolic fraction. The *n*-BuOH fraction was subjected to solid-phase extraction, and the resulting fractions were separated by reversed-phase HPLC, leading to the isolation of compounds **37 - 44**. In addition, the aqueous methanolic fraction was subjected to reversed-phase HPLC, resulting in the isolation of compounds **37-39** and **42-43.**
[32]	ND	The solid crude was dissolved in EtOH: H_2O and was partitioned to produce concentrated hexane, dichloromethane extract, and the aqueous solid. The aqueous partitioned extract was separated on the *octadecyl*-silica column by medium pressure resulting in 14 fractions (A to N). Fraction C from the aqueous partition was further purified using the ODS column by gravity liquid chromatography, yielding compound **45**. Fractions C and D of the aqueous partitioned extract were then combined and subjected to medium-pressure liquid chromatography using an octadecyl-silica column, resulting in compounds **46** and **47.**
[21]	The compounds were extracted with exhaustive baths of CH_3OH/CH_2Cl_2	The crude extract was initially partitioned between CH_2Cl_2/H_2O, producing aqueous and organic phases. The organic phase was concentrated under reduced pressure and partitioned between aqueous CH_3OH and hexane. The CH_2Cl_2 fraction was subjected to a Solid Phase Extraction with RP-18 using a gradient from H_2O to CH_3OH and then CH_2Cl_2. The fraction eluted with H_2O/CH_3OH was separated by RP-HPLC afforded compounds **48, 51,** and **52**. The aqueous methanolic fraction was submitted to a Solid Phase Extraction with RP-18 afforded compounds **50** and **53**.

[18]	The compounds were initially extracted with 96% EtOH and then with MeOH.	The extract was resuspended in MeOH and partitioned with hexane. The MeOH fraction was evaporated, suspended in EtOAc, and partitioned against H2O, resulting in an EtOAc fraction. The fractions were subjected to reversed-phase column chromatography on C8 cartridges, and eluted with a gradient of MeOH in H2O. Analysis by HPLC-PDA-MS indicated bromopyrrole alkaloids.
[27]	The compounds were extracted after the freeze-drying of the sample with 3 baths of MeOH/CH_2Cl_2.	The crude extract was subjected to silica gel chromatography and eluted with a gradient petroleum ether /EtOAc, yielding 11 fractions (A−K). Fraction F was further separated by CC on silica gel, eluting stepwise with petroleum ether/EtOAc, and resulting in six sub-fractions (Fr. F1−F6). Fr. F5 was separated by reversed-phase semipreparative HPL, resulting compounds **54, 55,** and **57.** Fr. H was chromatographed on an ODS column using a gradient elution of MeOH/H_2O to get eight final fractions (Fr. H1−H8). Fr. H3 was further chromatographed over silica gel, eluting with petroleum ether, resulting in five fractions (H3a−H3e). Fraction H3c was further purified with CH_3CN by HPLC, resulting in compound **56**. Fr. H5 was subjected to silica gel CC with petroleum ether, resulting in 8 sub-fractions (Fr. H5a−H5h). Fr. H5g was purified by semi-preparative HPLC, yielding compound **58.**

Table S2. Risk of bias assessment results based on ToxRTool assessment criteria

Reference	I	II	III	IV	V	Total	Reliability Categorization
[30]	4	2	0	1	0	7	Not Reliable
[16]	4	2	6	2	0	14	Reliable with restrictions
[22]	4	1	4	2	1	12	Reliable with restrictions
[24]	4	2	5	2	1	14	Reliable with restrictions
[25]	4	2	5	2	1	14	Reliable with restrictions
[23]	4	3	4	2	1	14	Reliable with restrictions
[26]	4	2	2	2	1	11	Reliable with restrictions
[28]	4	2	6	2	1	15	Reliable without restrictions
[19]	4	3	6	3	2	18	Reliable without restrictions
[17]	4	3	6	3	2	18	Reliable without restrictions
[31]	2	2	5	3	2	14	Reliable with restrictions
[20]	4	3	6	3	2	18	Reliable without restrictions
[32]	4	3	4	2	1	14	Reliable with restrictions
[21]	4	3	6	2	1	16	Reliable without restrictions
[18]	4	2	3	2	1	12	Reliable with restrictions
[27]	4	2	4	2	1	13	Reliable with restrictions
[29]	4	3	5	3	2	17	Reliable without restrictions

I: test substance identification; II: test system characterization; III: study design description; IV: study results documentation; V: plausibility of study design and data

Table S3 Synthesis of scientific evidence: GRADE

Outcomes	Limitations	Inconsistency	Indirectness	Imprecision	Publication bias	Trials	Quality of the evidence (GRADE)
**MIC**							
	✓	✓	✓	✓	✓	[30]	
	✓	✓	a	b	c	[16]	
	✓	✓	✓	b	c	[22]	
	✓	✓	a	b	c	[24]	
	✓	✓	a	b	c	[25]	
	✓	✓	a	b	✓	[23]	
	✓	✓	a	b	✓	[26]	
	✓	✓	✓	✓	✓	[28]	
	✓	✓	✓	✓	✓	[19]	
	✓	✓	✓	✓	✓	[17]	
	✓	✓	✓	✓	c	[31]	
	✓	✓	✓	✓	✓	[20]	
	✓	✓	a	b	✓	[32]	
	✓	✓	✓	✓	✓	[21]	
	✓	✓	a	b	✓	[18]	
	✓	✓	a	b	✓	[27]	
	✓	✓	✓	✓	✓	[29]	
							⊕⊕⊕

	MODERATE

✓: No Serious Limitations; [a]: negative controls in undescribed tests; [b]: poor statistical description; [c]: Absence of methodological information.

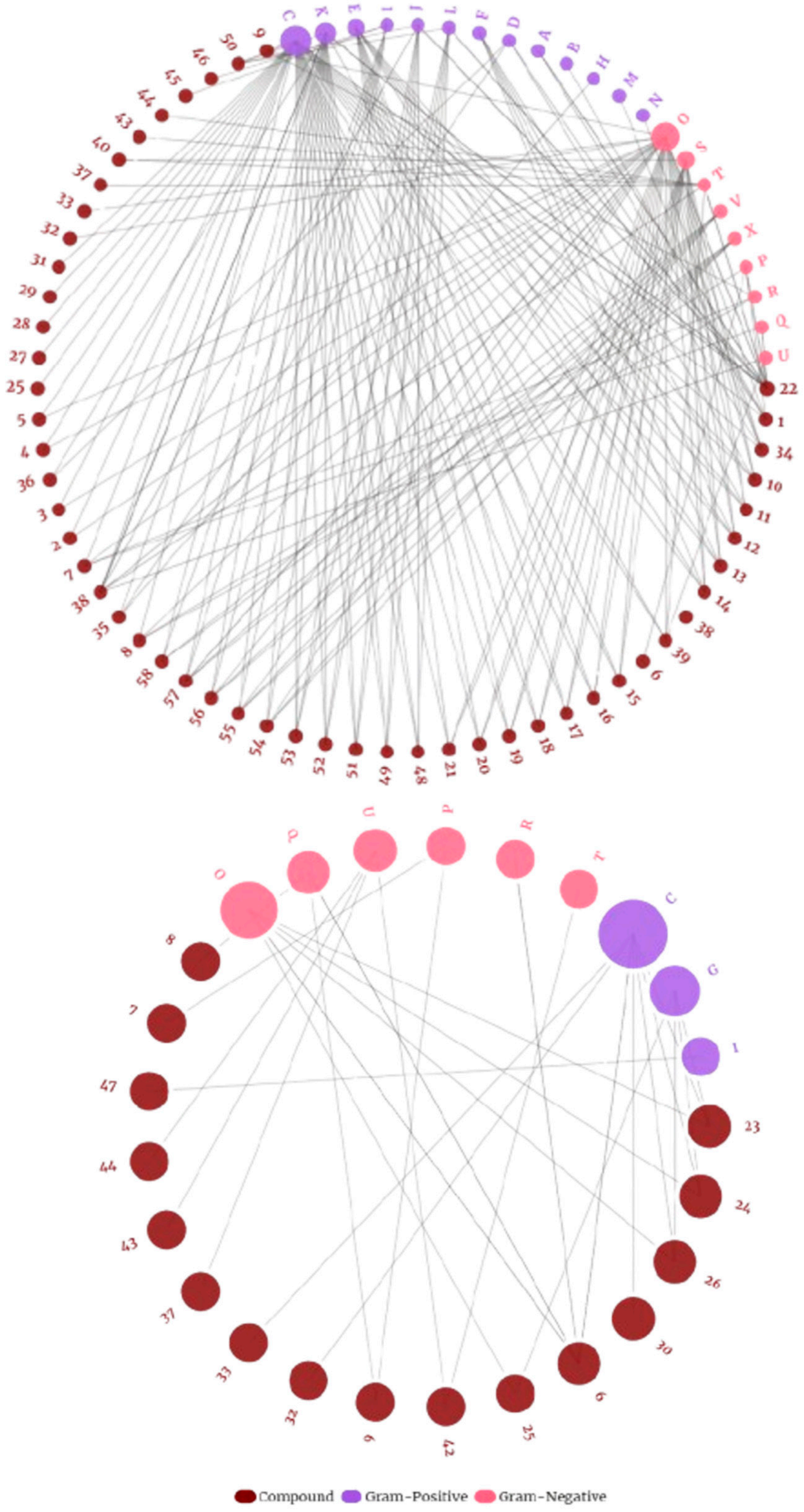

A

B

Figure S1 - Graphical representation of the compounds evaluated for their antibacterial activity against other bacterial strains, including Gram-positive and Gram-negative species. Among the Gram-positive strains, the most frequently used for the evaluation of this

antimicrobial activity were: *Staphylococcus aureus* [16,18,19,21–26,28–31]; *Enterococcus faecalis* [17,21,23,27]; and *Streptococcus pyogenes* [24,25]); conversely, among the gram-negative strains, the most extensively studied were *Escherichia coli* [16,18,22–27,30,31], *K. pneumoniae* [18,20,23], and *A. baumannii* [18,20].

A) Graphical representation of compounds that inhibit bacterial growth; B) Graphical representation of compounds that lack bacterial growth inhibition. In both graphical representations, each tested compound is linked to the bacterial strain used to evaluate its activity. Each letter stands for bacterial strain, while numbers stand for compounds. For example, A: *Streptococcus Faecalis*; B: *Bacillus Subtilis*; C: *Staphylococcus aureus*; D: *Bacillus cereus*; E: *Streptococcus pyogenes*; F: *Streptococcus faecium*; G: *Mycobacterium intracellular*; H: *Clostridium sporogeneses*; I: Streptococcus pneumoniae; J: *Enterococcus faecalis*; K: *Enterococcus faecium*; L: *Micrococcus Flavus*; M: *Listeria monocytogenes*; N: *Escherichia coli*; O: *Salmonella Typhi*; P: *Shigella flexineri*; Q: *Vibrio cholerae*; R: *Klebsiella oxytoca*; S: *K. pneumoniae*; T: *A. baumannii*; U: *Vibrio vulnificus*; V: *Vibrio parahaemolyticus*; X: *Enterobacter cloacae*; and **1:** Longanide; **2**: Haliclonacyclamine E; **3**: arenosclerins A; **4**: arenosclerins B; **5**: arenosclerins c; **6**: Purealidin Q; **7**: Purpurealidin B; **8**: 16-Debromoaplysamine-4; **9**: Purpuramine I; **10**: (R)-6''-Debromohamacanthin A; **11**: (R)-6'-Debromohamacanthin A; **12**: (S)-6''-Debromohamacanthin B; **13**: trans-3,4-Dihydrohamacanthin A; **14**: cis-3,4-Dihydrohamacanthin B; **15**: (S)-6',6''-Didebromohamacanthin A; **16**: (R)-6'-Debromohamacanthin B; **17**: (R)-6',6''-Didebromohamacanthin B; **18**: (3S,5R)-6''-Debromo-3,4-dihydrohamacanthin B; **19**: (3S,6R)-6'-Debromo-3,4-dihydrohamacanthin A; **20**: Spongotine B; **21**: (3S,5R)-6'-Debromo-3,4-dihydrohamacanthin B; **22**: 2-(2',4'-dibromophenoxy)-4,6-dibromophenol; **23**: (-)- ageloxime D; **24**: (−)-8′-oxo-agelasine D; **25**: (-)- ageloxime B; **26**: (+)-2-oxo-agelasidine C; **27**: Ianthelliformisamine A; **28**: Ianthelliformisamine B; **29**: Ianthelliformisamine C; **30**: Aplysamine 1; **31**: Araplysillin I; **32**: Svl1; **33**: Svl2 ; **34**: Avarol; **35**: halistanol sulfate; **36**:halistanol sulfate C; **37**: Ageliferina; **38**: Ageliferin B; **39**: Ageliferin D; **40**: Sceptrin; **41**: Nakamuric acid; **42**: 4-Bromo-1H-pyrrole-2-carboxylic Acid; **43**: 4,5-Dibromopyrrole-2-carboxylic acid; **44**: 3,7-Dimethylisoguanine; **45**: aaptamine; **46**: isopentylamine; **47**: Tyramine; **48**: (+)-8-epiagelasine T; **49**: (+)-10-epiagelasine B; **50**: (+)-12-hydroxyagelasidine C; **51**: (+)-ent-agelasine F; **52**: (+)-agelasine B; **53**: (+)-agelasidine C; **54**: Phyllospongiane A; **55**: Phyllospongiane B; **56**: Phyllospongiane C; **57**: Phyllospongiane D; **58**: Phyllospongiane E.
